# PlayDoc M.D.: Sexual Harassment and Discrimination in US Medical Schools in the 1960s and 1970s

**DOI:** 10.1093/shm/hkae044

**Published:** 2024-07-26

**Authors:** Elizabeth Evens

**Affiliations:** Medical School, Newcastle University, Newcastle, UK

**Keywords:** sexual harassment, gender, sexuality, medical school, twentieth century

## Abstract

Since women’s entrance to the historically male-dominated medical profession in small numbers in the nineteenth century, they faced numerous exclusions and obstacles. In the 1960s and 1970s, as the number of women attending co-educational medical schools increased significantly, male students and faculty members responded with renewed opposition by deploying hypersexualised innuendo including references to *Playboy* magazine. This article brings together a range of material, including *Playboy*, student yearbooks, teaching materials, contemporary studies and oral histories, to document the masculine heterosexual peer culture that pervaded US medical schools, where sexual innuendo and centrefold-style images were commonplace. This learning environment influenced male students, perpetuating harmful views about women and fostering camaraderie at the expense of their female colleagues. These experiences also impacted female students, who confronted and negotiated encounters with sexual harassment, while balancing study, career ambitions and personal wellbeing.

In the landmark sociological study at the University of Kansas Medical School in the 1950s, *Boys inWhite*, Howard S. Becker described the process of ‘boys becoming medical men’ as they learned their instructors' values and developed a shared student culture through study, socialising, assessments and clinical experience.^1^ Although small numbers of women had graduated from medical school since the nineteenth century and Kansas since 1906, the 1961 study described a ‘homogenous body’ of white male students, which was replicated at other schools across the country. This changed in the two decades following Becker's study as increasing numbers of women enrolled in co-educational medical schools, becoming an unmissable presence in lecture theatres, anatomy labs, on the wards, amongst the faculty and in the profession. Women’s presence not only changed the educative experience, but also the masculine identity that bound the ‘boys in white’ throughout their careers. In the words of Dr Bernadine Healy, a white woman who attended Harvard Medical School in the 1960s, women entering this environment were made to feel like ‘oddities and outsiders…invading a world that inalienably belonged to men'.^2^ Drawing upon medical school yearbooks and textbooks, contemporary research and protests mounted by female activists, alongside student recollections in oral histories, this article documents how some male students and faculty responded to these changes with a backlash of misogyny. As part of this backlash, men invoked the sexual content, visual language, values and humour of *Playboy* magazine in an attempt to restate traditional gendered hierarchies within society and healthcare. This behaviour formed part of the sexual harassment perpetrated in medical schools and hospitals, which affected female students’ learning, careers and wellbeing.

Since women’s entrance to the professionalised field of ‘regular’ medicine in the mid-nineteenth century, women endured discrimination and hostility from male doctors. As historians have shown, women doctors forged gendered meanings of medical authority to claim a place in the exclusive field.^3^ In 1849, Elizabeth Blackwell became the first woman to receive an M.D. and thereafter small numbers of women graduated from historically male medical schools and new institutions for women, such as the Woman’s Medical College of Pennsylvania (WMCP). Despite these opportunities, obstacles persisted; women were underrepresented or excluded from medical school student bodies and faculties, professional associations, and internship and residency programmes. Medical men’s hostility could include harassment. In an infamous 1869 episode at Pennsylvania Hospital, over one hundred male medical students harassed a cohort of WMCP women attending a co-educational lecture through ‘yells, hisses, “cat-er-waulings,” mock applause, [and] offensive remarks upon personal appearance’.^4^ This hostility persisted as the number of women in medicine grew to comprise 6 per cent of doctors in 1910. Thereafter, the proportion of female doctors fluctuated between 4.5 and 6.5 per cent, before rising significantly in the 1960s.^5^

Historians have looked to female physicians as a case study to explore gender and professionalisation in the late nineteenth and early twentieth centuries, however the 1960s and 1970s remain an important, understudied period of profound change.^6^ In these decades, the number of female M.D.s significantly increased: women were 9.3 per cent of medical college matriculants in 1965 and 28.7 per cent in 1980.^7^ Informed by the expansion of higher education following World War Two and women’s liberation activism, in 1970 the Women’s Equity Action League (WEAL) filed a lawsuit against every medical school in the US alleging discrimination.^8^ Then in 1972, Congress passed Title IX as part of the Higher Education Amendments Act, authored by Representative Patsy Mink. Mink, who was Japanese American, was the first woman of colour to serve in Congress and had herself been rejected from twenty medical schools when she applied in 1942.^9^ As a result of this legislation, many medical schools became co-educational or began to admit significant numbers of women for the first time.^10^ The majority of this new cohort of students were white, middle-class women. Black doctors continued to be underrepresented in medicine and before 1968, the 2.5 per cent of doctors who were African American were typically graduates of Howard University College of Medicine and Meharry Medical College.^11^ Black women doctors were acutely underrepresented. In the 1970 census, Black women accounted for 1,051 of the total 20,824 women doctors documented.^12^ Civil Rights affirmative action programmes, the expansion of student finance through the 1965 Higher Education Act, and student activism led more Black students to enrol in historically white institutions in the 1970s.^13^

At the same time as shifting medical school demographics changed medicine from the inside, the feminist women’s health movement challenged the profession from the outside. Building upon women’s liberation protests of the 1960s, feminist academics and activists argued that gendered hierarchies and prejudices shaped the US healthcare system. They showed that medicine privileged male doctors’ expertise over female patients’ experiences, manifesting in the mistreatment, misdiagnosis and endangerment of female patients. Female medical students were a reminder of the challenges male students and faculty faced from without as well as within. New anxieties stemming from women’s increased presence in the classroom transformed student spaces into crucial sites of masculine heterosexual performance, where sexual innuendo fostered homosocial comradery, oftentimes at the expense of female students. Contemporary activists identified this behaviour as a backlash. Dr Mary Howell, the first female dean at Harvard Medical School from 1972 to 1975, described a phenomenon where one or two women in a cohort were ‘tolerated and thought well of’, but as medical men started ‘seeing a lot more women around…they weren’t seen as pets [any longer]’ but rather became threatening. Howell believed that this ‘engendered a kind of increased conservativism’ among male students, which included the incorporation of hypersexualised tropes into longstanding hostility towards women in medicine.^14^

This article begins by exploring the gendered dynamics of the hierarchical medical world through an examination of *Playboy* magazine, which offered commentary on medical matters in centrefolds, cartoons and articles. This analysis accounts for how *Playboy’s* philosophies of masculinity and sexuality came to pervade medical education. Next, I demonstrate how women faced sexual harassment and gender discrimination during medical school. Male faculty members and students invoked *Playboy* to express their view of women and to make female students uncomfortable, suggesting that women’s place in the classroom was not as learners, but rather as objects of men’s heterosexual gaze. The concluding section of the article considers women's diverse responses to this harassment. It details how women led public campaigns against this discrimination, challenged men’s behaviour in everyday interactions, or attempted to ignore or minimise the impact of this behaviour, as they balanced their mental health, interests and personal life, all while pursuing a demanding medical degree.

## Gender, Sexuality and Medicine in the Pages of *Playboy* Magazine

Founded in December 1953 by former Esquire writer Hugh Hefner, *Playboy* was a huge cultural event. The magazine reached an enormous audience, peaking at seven million in 1972, but *Playboy*’s visibility and influence exceeded this subscription base.^15^ Historians have pointed to the publication as emblematic of the changes of the 1950s and 1960s, including the rise of the consumer society, neoliberalism and the sexual revolution.^16^ In addition to the famous centrefold photographs of nude women, issues included articles, reader correspondence and interviews with diverse and influential figures. Written and visual material focused on hyperfeminised, sexualised women, reflecting Hefner’s ‘*Playboy* Philosophy’ that championed sexual freedom for women predicated on their adherence to gender norms.^17^ As historian Julie Willett showed, *Playboy*’s ‘male chauvinist’ attitude to women and female sexuality extended beyond the pages of the magazine to influence contemporary stand-up comedy, television and films and consumer goods.^18^ In addition to commercial gain, men could weaponise the *Playboy* Philosophy and its ideology of female sexuality to target women within work and educational environments.

Historians have highlighted the multiple meanings of *Playboy* for women’s rights in the 1960s and 1970s. For many second-wave feminists, *Playboy* epitomised women’s sexual objectification.^19^ However, historian Carrie Pitzulo described a more nuanced legacy of *Playboy*, pointing to the magazine’s promotion of women’s right to sexual pleasure, access to birth control and financial support for abortion rights and the American Civil Liberties Union.^20^ Nonetheless, as Elizabeth Fraterrigo highlighted, *Playboy* galvanised many contemporary feminists, who saw it as typical of exploitative portrayals of women in the media and consumer culture.^21^ Other feminists, including journalist Gloria Steinem, focused on the discriminatory employment practices and sexual harassment within *Playboy* Clubs.^22^ But it was not solely feminist activists, lawyers and journalists who contested the meanings of *Playboy*; this article provides a new perspective on these issues by showing how women encountered and challenged the magazine’s influence in the realm of medical education.

Like *Playboy*'s commentary on contemporary feminism, the magazine's exploration of the medical world formed part of its engagement with middle-class masculinity. Through articles, centrefold images and cartoons, *Playboy* frequently looked to medicine as a source of comedy and titilation, so much so that in 1971, 158 medical themed cartoons from the magazine appeared in a book, *Doctor! Doctor!*.^23^ Cultural depictions contributed to the meanings of being a doctor for professionals, patients, and the public. In the mid-twentieth century, hospitals proved a popular setting for television serials and novels. Romantic fiction publisher Mills & Boon commissioned former nurses to author their popular doctor–nurse romance novels, which formed one quarter of their total sales by 1957.^24^ At this time, several popular television shows including *Ben Casey*, *Dr Kildare*, *General Hospital*, *Julia* and *Marcus Welby* dramatised the hospital environment; the American Medical Association even consulted on some of these serials’ scripts.^25^ Meanwhile, doctors published popular autobiographical accounts of medical training, such as the anonymous Doctor X’s 1965 *Intern* and William A. Nolan’s 1970 *Making of a Modern Surgeon.Playboy* too sought to capitalise on professionals’ and consumers’ interest in depictions of the inner workings of a hospital.

This media and professional interest in representations of medicine perhaps explains why Professor of Psychiatry Dr Harold I. Kaplan wrote a letter to the *Playboy Forum* in December 1971 about women doctors. The Forum published readers’ letters, with the aim of furthering editorial viewpoints or generating controversy, as Elizabeth Fraterrigo explained.^26^ Kaplan’s letter fulfilled the latter goal, sparking a divisive exchange of correspondence. Kaplan had surveyed medical schools and found a ‘disinterest or overt hostility to women in medicine’. He quoted two deans, one said: ‘I just don’t like women—as people or doctors—they belong at home cooking or cleaning’, while another replied, ‘I have enough trouble understanding my wife and daughter—I certainly don’t want such people as medical students’.^27^ Kaplan’s letter inspired a series of replies including from Dr Frances S. Norris. Norris expanded upon these findings, linking them to contemporary feminist health politics. She explained that these deans’ responses spoke to ‘patriarchal attitudes within medicine’s centres of power and policy’ that affected female patients as well as clinicians. She cited insufficient education about the side effects of contraceptives, unnecessary surgeries and communication failures that undermined informed consent.^28^

Additional replies published in the *Playboy* Forum show the centrality of sexuality and reproduction to the debate over women’s place in the profession. College student, Mary Marvin Johnson wrote to the forum detailing the hostility she faced when applying to medical school. She recounted a double standard whereby, ‘Married as well as single males are accepted into medical schools’, meanwhile ‘schools reject single women because they may marry and married women because they may get pregnant’. She surmised that ‘It seems as if males have their sexual freedom, whereas a woman medical student is almost forced to take a vow of celibacy’.^29^ A subsequent letter from Dr John W. Docktor of the University of Illinois Hospital unironically confirmed her impression, arguing that Norris and Johnson ‘fail[ed] to recognize the merit of discrimination’.^30^ He opined that, ‘When women stay in the profession and have their babies, they impose severe hardships on their male colleagues.’ Docktor’s solution was to impose reproductive restrictions; 'I think women in medicine should take two years out…and try to have their families…after the two years, women doctors should have their tubes tied'.^31^ Docktor, and other men in the profession, grounded their opposition to the rising number of women doctors in arguments about gender, reproduction and work. This fraught exchange of letters shows how the corresponding medical professionals and *Playboy*’s readership considered the status of the woman doctor and her sexuality as a topic of public commentary.

Aside from these letters, *Playboy* rarely addressed women doctors, instead focusing on two other women in medicine: nurses and patients. *Playboy* selected a number of white women who worked as allied medical practitioners as their ‘Playmate of the Month’, drawing upon the visual language of nursing and invoking the ‘naughty nurse’ trope.^32^ As Elizabeth Fraterrigo argued, *Playboy* cartoons’ main intent appeared to be sex and nudity, but the punchline and exaggerated pictorial humour relied upon broader cultural tensions and assumptions. In these medical themed illustrations, male patients imposed unwanted sexual advances upon nurses. In a November 1968 cartoon, a woman arrived at the hospital asking ‘Excuse me, nurse, can you tell me where my husband--’, when she is seemingly cut off by the sight of a white woman fleeing a hospital room with bedraggled hair, a tattered nursing uniform and a shredded cap. The wife remarked ‘Oh, never mind’, having found her husband based on the nurse’s appearance. An October 1969 cartoon repeated this joke. It showed a woman reading a hospital invoice at her husband’s bedside, remarking ‘what’s this item for the two torn nurse’s uniforms’.^33^ These cartoons implied that male patients had sexually assaulted nurses, eliding the realities of this violation for the nurses, instead focusing on the patient’s wife’s discovery and familiarity with their husband’s behaviour, which they presented as a source of humour.^34^ These jokes targeting female healthcare providers and trivialising sexual assault also featured in medical students’ invocation of *Playboy*, as the next section will address.

Several *Playboy* cartoons depicted male doctors exploiting the intimacies of physical examinations to leer at or manipulate female patients. These cartoons presented these scenarios as moments of erotic possibility to excite male readers, rather than incidents of malpractice and exploitation. An April 1965 cartoon showed a nude female patient positioned in front of a large desk, behind which a male doctor sits alongside three men in suits, peering at the woman. The caption read ‘they’re not exactly consulting physicians Mrs Walters. As a matter of fact, they’re just some fellows I play golf with’.^35^ Many other cartoons similarly depicted a female patient who visited a doctor in search of medical advice, but instead received his romantic attentions. While the illustrations suggested female patients’ naivety to titillate readers, they also captured the reality of women’s potential vulnerability; pictured male physicians objectified female patients, all the while ignoring their health concerns. Unlike contemporary feminist women's health activists, these cartoons invited the reader’s amusement, rather than condemnation. *Playboy* cartoons dramatised men’s dual identities as professionals and sexual actors in a variety of occupational settings, but the medical themed images drew inspiration from, and sexualised, physical and emotional intimacies between doctors and patients in clinical settings. The next section addresses how medical school education material and yearbooks likewise merged male students and staffs’ erotic and professional gazes, with profound consequences for female students navigating this environment.

## Teaching, Peer Culture and Sexual Harassment at Medical School

Scholars tracing women’s entrance to male-dominated fields in the late nineteenth and early twentieth centuries have shown how gender ideologies were both a liability and a resource. Women had to claim professional skills and status that were traditionally gendered masculine, while navigating accusations of becoming mannish or unsexed.^36^ In this environment, some women doctors claimed a gendered authority to impart scientific truths and extend a homosocial sympathy to female patients, as historian Regina Morantz-Sanchez has argued.^37^ Meanwhile, according to Kim Girouard and Susan Lamb, other women doctors minimised their femininity to claim a serious, rigorous authority gendered masculine in an attempt to be accepted by the male establishment.^38^ In the 1960s and 1970s, male doctors and students continued to subject their female colleagues to longstanding forms of discrimination, including allegations that women were unable to practice medicine due to marriage and childrearing. However, in this period, hypersexualised centrefold-style images and questions about contraception became a new means to restate gender differences in the hierarchical medical world as the composition of the profession changed.

In the 1961 study ‘Boys in White’, Becker described the medical school interview as a moment when teachers ‘look at [applicants] seriously and anxiously’ to ‘ask themselves and one another, "Will this bright boy really make a medical man?"'; for female applicants, this was an impossible question to answer.^39^ Several women doctors recalled how interviewers stated that there were quotas for admitting women. In the 1960s, one interviewer told Dr Carolyn Parry Decker, a white woman from Pennsylvania, that ‘this interview is really a waste of time because we already have our quota of six women’.^40^ Other women doctors recalled being informed that there were seperate quotas for Black applicants and female applicants; one Black woman doctor was told ‘you will take up two slots if we admit you’.^41^ In addition to facing quotas, many women doctors recalled confronting gendered hierarchies within healthcare during the admissions process. A male doctor at St. Louis University Medical School told Dr Nadine Bruce: ‘Your grades are okay, but you’ve gone to a small girls’ school. You’re nothing special, so why don’t you just go and be a nurse?’ In response to the interviewer, Bruce quipped ‘if your foot itches, you don’t scratch your shoe’.^42^ Other interviewers subjected female applicants to intrusive questions about their reproductive health. During an interview at Ohio State University College of Medicine, Dr Susan Benes sat opposite nine men who ‘grilled [her] the entire time about what form of contraception [she] was using’ and asked her to ‘promise not to get pregnant while [she] was in medical school’.^43^ Meanwhile Dr Bernadine Healy recalled being asked if her mother was ‘menopausal and therefore frustrated’ and if this had ‘inspired [Healy’s] clearly deviant life choice’ in applying to medical school.^44^ Women’s very presence at medical school interviews problematised traditional gendered hierarchies and roles within medicine, and interviewers responded through inappropriate questions about their sexual and reproductive lives.

Even after gaining hard-fought admission to medical school, lecturers, hospital staff and patients could perpetuate sexist and racist cultures of exclusion that could alienate women and limit their learning opportunities. For Black women, racialised and gendered discrimination impacted their experiences of training. For example in an oral history, Dr Vanessa Gamble—a Black woman from Philadelphia—recalled introducing herself as a student doctor to a male patient, who did not believe her. Instead, the patient insisted that Gamble was a domestic worker and repeatedly asked her colleagues why she had not cleaned his room. In another encounter, a white nurse refused to believe that Gamble was a student physician and took a set of medical notes from her. Gamble summarised that the nurse ‘saw a Black woman, and it didn’t equal medical student’.^45^ Through these misidentifications, patients and other professionals expressed gender and racial biases about women’s roles and skill levels, and, as Gamble’s experiences show, Black women could face distrust from colleagues and patients. These encounters significantly impacted students’ and doctors’ experiences of the workplace, in addition to limiting learning opportunities.

Just as episodes of misidentification isolated women from the doctor role, teaching that courted male heterosexual desire also isolated women from their peer group. Dr Gamble recalled female students hissing when a professor began a lecture by saying 'medicine is the profession that seperates the men from the boys'.^46^ Other lecturers used sexualised images in teaching. Dr Stephanie Woolhandler, a white woman graduate of the Louisiana State University of Medicine, listed misogyny experienced as a student in the 1970s as her biggest career obstacle. She recalled breast anatomy lectures ‘illustrated with *Playboy* centrefolds’ and an atmosphere in which ‘sexist jokes and sexual propositions from professors were common’.^47^ Other students also recalled lecturers’ use of these images in teaching. In 1973, Mary Howell—a white woman and the first female dean at Harvard Medical School—surveyed over 146 female medical students from 41 schools nationwide about their experiences. In their responses, female students referenced pin-up photographs in lectures more than a dozen times.^48^ In her analysis of these responses, Howell argued that these ‘laugh-getting’ images formed part of a hypersexualised culture that knitted medical and sexual content together, where ‘comments about female sexual practices, habits, and preferences [were]…given as embroidery overlaid as factual material’. Echoing *Playboy* cartoons, instruction on physical examinations, anatomy and physiology sexualised information about the body to titillate a presumed male audience. For Howell, this teaching promoted a ‘men’s club’ atmosphere and the assumption ‘that any man has the right to regard any woman—colleague or patient—as an object of sexual interest’.^49^ One respondent outlined her belief that professors used these jokes to build comradery and affection between peers and their instructors; community at the expense of, and excluding, women. As the student explained, jokes fostered male homosocial bonding, forging networks that could facilitate members’ professional advancement, while promoting negative attitudes towards female patients and their health, in addition to isolating female students.

These educational materials built comradery between white male students while adversely impacting marginalised students. In an interview about three generations of Black women in medicine in her family, Dr Sharon Brangman recalled how centrefold images featured in her education at New York Upstate Medical University, Syracuse, in the late 1970s.^50^ According to Brangman, a white male lecturer who displayed centrefolds in anatomy instruction also used photographs of a Black athlete and imitated African American Vernacular English when teaching about muscles.^51^ Such behaviour marginalised Black students directly, while also promoting a discriminatory attitude among the white medical students in attendance. Brangman recalled how this professor would also stand behind Black students as they took tests and blow smoke from a pipe over them. Experiencing secondhand smoke and unwanted physical proximity could be at once harmful to students’ health, while also potentially influence their examination performance.^52^ Professors’ choice of teaching materials and conduct could make Black students uncomfortable, while endorsing such behaviour and values among their white peers.

Instructors’ selection of images to illustrate anatomy harmed marginalised students directly, while promoting gendered and racialised knowledge that could influence how students treated patients. In 1971, Duke University Professors Frederick Becker, James Wilson and John Gehweiler authored a textbook entitled *The Anatomical Basis of Medical Practice*, which used pin-up photographs of naked white women to illustrate surface anatomy. *Playboy* centrefold photographer Peter Gowland supplied the images for the textbook, which received positive reviews in the *Lancet* and *Journal of the American Medicine Association*. In the preface, the authors addressed their decision to include these images; explaining that ‘only on rare occasions will the attractive, well-turned specimen appear before [the doctor] for consultation’ and ‘he should be prepared for this pleasant shock’. They continued, ‘sorry that we cannot make available the addresses of the young ladies who grace our pages. Our wives burned our little address books’. This introduction and inclusion of sexualised images of women presented male heterosexual desire as a prescriptive part of the medical student identity and encouraged students to sexualise female patients.^53^ In the introduction, the authors described the ‘female models’, who all had white skin, as ‘model females’. As Amanda Friz and Marissa Fernholz highlighted, the textbook 'link[ed] the aesthetics of White standards of beauty to the medical standards of White bodies as universal human bodies', creating biased perceptions of ‘normal’ anatomy that contribute to and perpetuate racialised healthcare inequalities.^54^

Contemporary activists drew connections between medical textbook images and the treatment of patients. Medical women mobilised to protest the authors’ use of pin-up photographs in the textbook. Dr Estelle Ramey, a physiology and biophysics professor at Georgetown School of Medicine and head of the Association of Women in Science, led this campaign.^55^ Speaking before Congress in the hearings for the 1974 Women’s Educational Equity Act, Ramey cited the textbook as emblematic of the discrimination women faced in medicine and branded it a product of the ‘adolescent fantasies of the authors’. Ramey connected the images to the treatment of female patients; arguing that more troubling than these photographs, were the harmful myths about women that appeared throughout medical literature. She cited the common references to female patients as ‘specimens’ and myths about female sexuality, hormones and mental health.^56^ For Ramey and others, visual representations were connected to the real experiences of women in the medical world, because they gave medical students inaccurate expectations and understandings, which negatively impacted patient care.

In addition to promoting biased treatment among students, professors’ conduct could empower male students’ hostility towards their female classmates. In response to Harvard dean Mary Howell’s survey, one student recalled that lecturers made 'little jokes, invariably with women as the butts, in an attempt to gain rapport’. They reported that this ‘encouraged the men in my class to adopt a similar attitude’ and when women pointed out these ‘humiliations, they were further belittled’.^57^ As cardiologist Dr Bernadine Healy described, ‘future doctors were being rewarded by their teachers for expressing their interesting attitudes toward women’.^58^ Many medical schools produced yearbooks that captured the dominant peer culture of the cohort, which included sexual innuendo, images of naked women and references to *Playboy*. Yearbooks both reflected and constructed the student experience; capturing memories of people and events, but also creating meaning to bond both the cohort and, in the case of medical schools, a profession.^59^

**Fig. 1:**
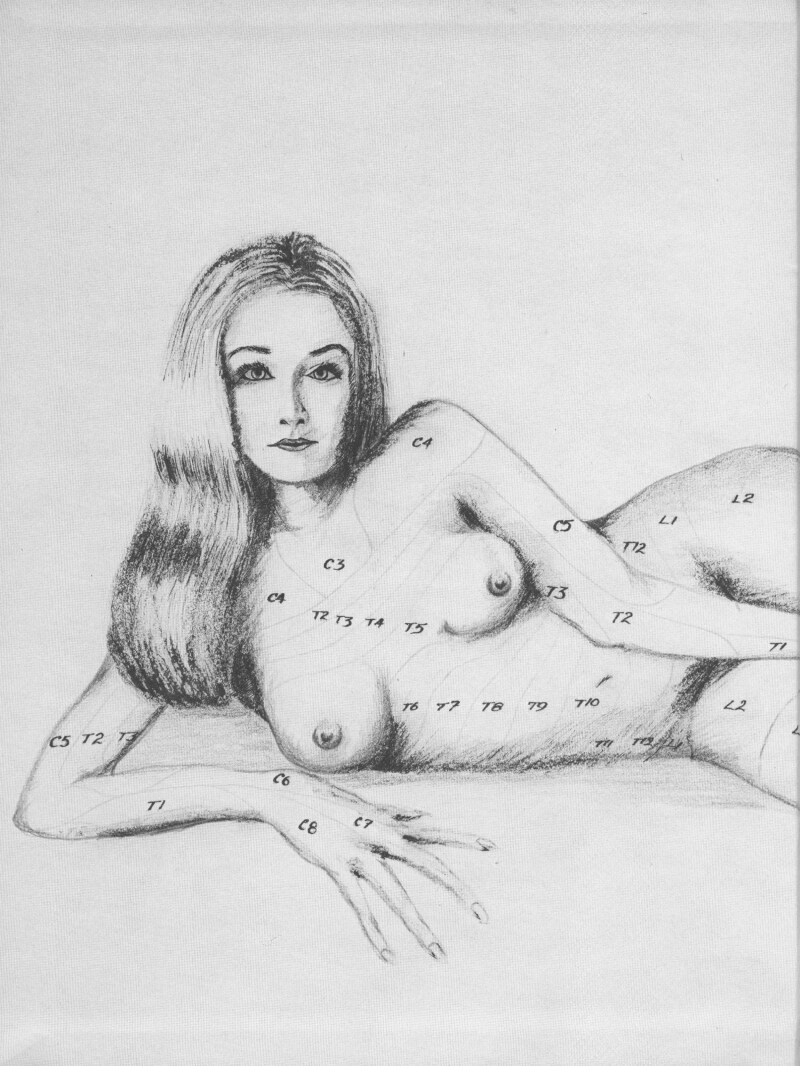
Illustration of a nude woman labelled with the dermatomes, University of Utah School of Medicine yearbook 1973 ‘Medicine Man’ yearbook. Spencer S. Eccles Health Sciences Library, University of Utah, Salt Lake City, Utah.

Yearbooks spliced 1960s and 1970s hypersexualised imagery with longstanding gendered medical hierarchies. Like *Playboy* cartoons, this visual material foregrounded nudity, but also drew upon broader medical themes. For instance, the 1973 University of Utah School of Medicine yearbook opened with a centrefold-style sketch of a nude white woman labelled with ‘C3’, ‘T2’ and ‘L5’ as shown in Figure 1. These labels were a map of the dermatomes, areas of the skin innervated by a particular spinal nerve, which medical students were expected to learn in order to test sensation during neurological examinations. The image thus weaved specialised medical knowledge with sexualised imagery to titillate male medical viewers. Another series of photographs in this yearbook showed a nude woman demonstrating three medical skills; ‘inspect’ holding an ophthalmoscope, ‘palpate’ while clutching her breast, and ‘auscultate’ using a stethoscope on herself.^60^ The illustration and photo sequence were reminiscent of *Playboy* cartoons and likewise confirmed women’s position as objects, rather than holders, of medical knowledge. The inclusion of these photographs alongside family portraits of the graduating class reflected how this material formed a mainstream part of medical education in this period.

**Fig. 2:**
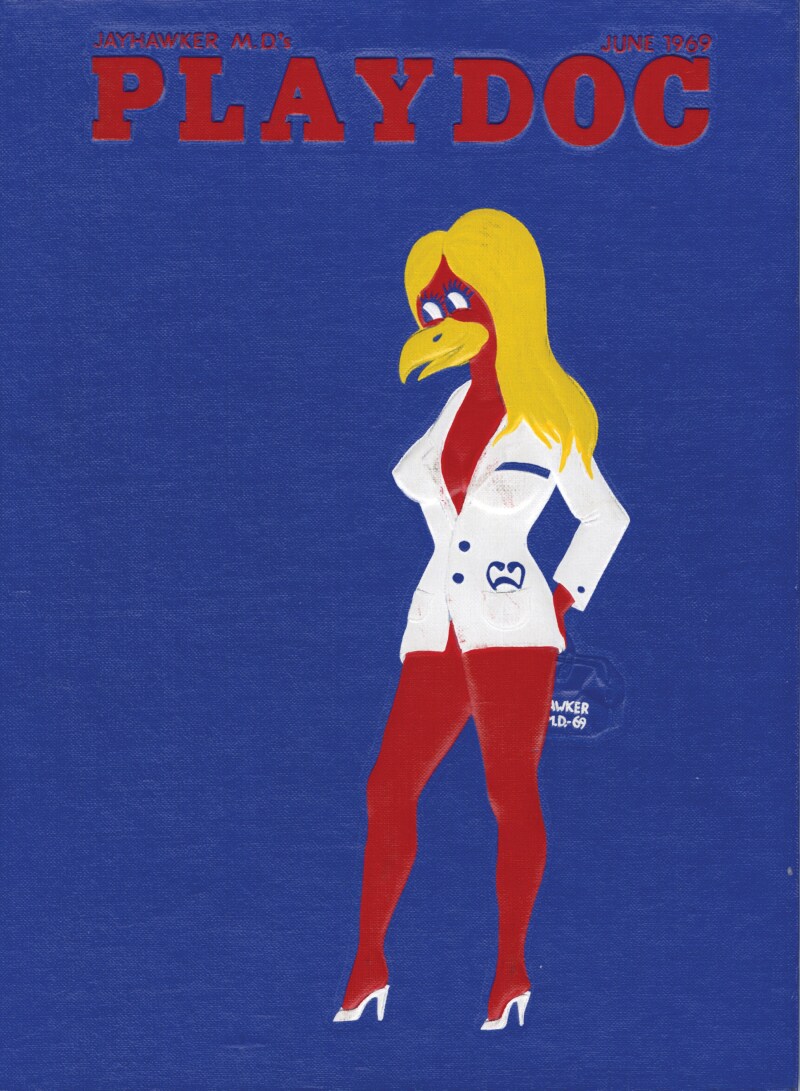
The illustrated cover of the 1969 ‘PLAYDOC’ yearbook from the University of Kansas School of Medicine showing an anthropomorphised, feminised and sexualised version of the university’s Jayhawker mascot with blonde hair and a white coat. Image courtesy of the University of Kansas Medical Center Archives, Kansas City, Kansas.

Medical yearbook editors frequently applied the visual vocabulary of *Playboy*—including bunnies and centrefold images—to nursing. The University of Kansas’ 1969 yearbook adopted an entire *Playboy* theme; it was entitled *PlayDoc* and featured a sexualised, pin-up version of the college’s jayhawker mascot on the cover as shown in Figure 2. The section of nurses’ yearbook photos was entitled ‘Bunnies’ and editors surrounded nurses’ photographs with the infamous rabbit logo.^61^ Similarly, the 1969 yearbook from Jefferson Medical College included a centrefold photograph of a white woman, who was nude except a white nurse’s cap. The model, who was not identified as a student, posed contemplating the ‘Repair of Cystocele’, a form of vaginal surgery illustrated through anatomical illustrations of the vagina. This served to heighten the sexualised depiction and synonymised the ‘nurse’ with female reproductive anatomy. Editors countered the increasing numbers of graduating female M.D.s listed as classmates in yearbooks, by sexualising women’s traditional role within medicine.^62^

In contrast to hypersexualised depictions of nurses, some student media used sexuality in a different way to criticise women doctors on the faculty. Dr Estelle Ramey described this stereotype of a female doctor as ‘a horse-faced, flat-chested female in supphose [stockings] who sublimates her sex starvation in a passionate embrace of the *New England Medical Journal*’.^63^ One oral history described a ‘fussy old spinster anatomy professor’, while another female doctor recounted ‘two female faculty members in the entire medical school, and they were tough old bi***hes—worse than the men’.^64^ One yearbook invoked *Playboy* to caricature female members faculty in this way. The 1969 Columbia University College of Physicians and Surgeons yearbook captioned a candid photograph of a middle-aged female faculty member wearing a lab coat, ‘Playmate of next month’.^65^ The editorial team used the language of *Playboy* to create humour at the woman doctor's expense and to suggest the age- and profession-dictated limits of female sexuality.

An episode at Harvard Medical School dramatically demonstrated these bifurcated depictions of medical women's sexuality. In an oral history interview, psychiatrist Dr Judith Herman, a Jewish woman from New York, described how female faculty members 'were so ridiculed' in the annual tradition of the Second Year Show. According to the psychiatrist, these women were depicted as ‘castrating monsters’, portrayed by female medical students wearing a ‘big lab coat and sensible shoes’. This uniform of serious scientific study obscured the students wearing it until, at a key moment during the play, they removed their coats to reveal ‘red satin’, while other women wore bunny costumes.^66^ Herman later recalled that she ‘wasn’t comfortable with the way the women were treated’ and that the attitude portrayed in these skits extended into the broader medical school environment; 'There was a bunch of [male students] who were just frankly hostile. Some faculty too–I mean, they would tell sexist jokes and use cheesecake pictures and the standard thing, making very demeaning comments about female patients'.^67^ Another woman doctor recalled that as a medical student, she was expected to tolerate similar inappropriate sexual behaviour, or be asked ‘Can’t you take a joke?’, and thus felt constantly caught between being a ‘“bi**h” and a “cutie”’.^68^ These recollections and the Harvard Second Year Show indicated the balancing act medical women had performed since the nineteenth century, navigating the perception of being seen as feminine and thus unprofessional, or professional and thus unfeminine; a dynamic that was recast with sexual innuendo in the 1960s and 1970s.

**Fig. 3:**
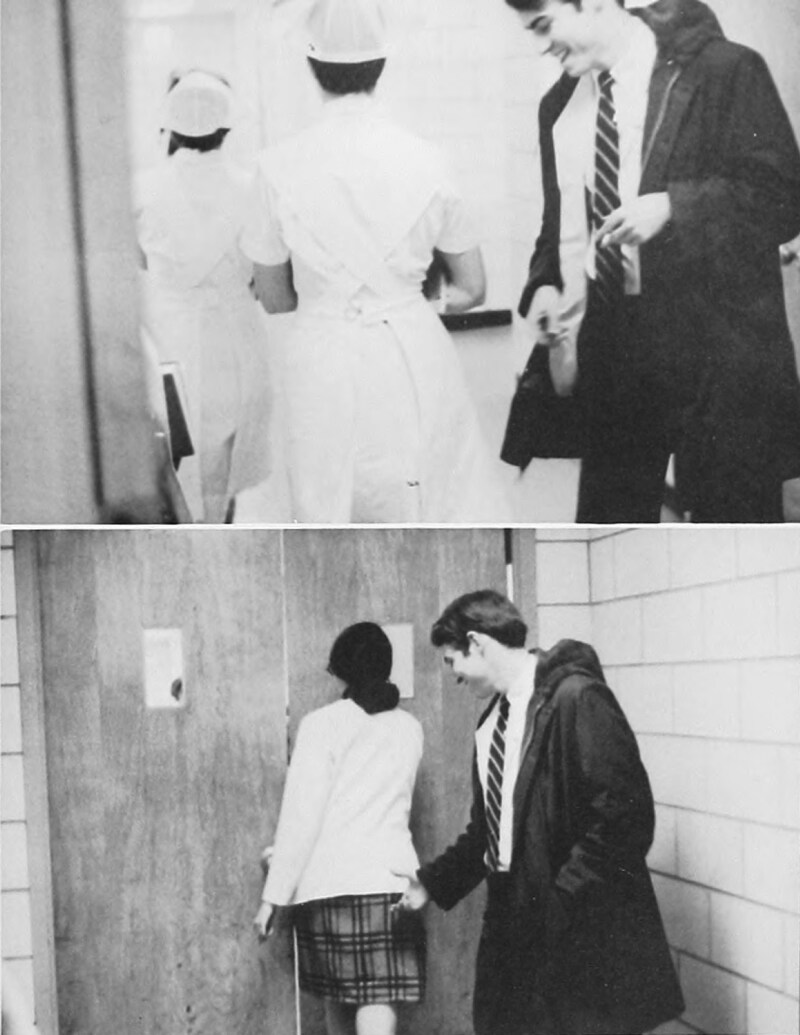
Image showing a male medical student simulating a sexual assault of a female nurse and a female medical student, captioned ‘Hunting: Chuck Jackson’ on the ‘Class Athletes’ section of the 1969 Columbia University College of Physicians and Surgeons yearbook. Archives & Special Collections, Columbia University Health Sciences Library, New York City, New York.

Other yearbook material showed how sexual innuendo could include implied sexual harassment and assault, much like the *Playboy* cartoons. Figure 3 is a series of images from the 1969 Columbia University College of Physicians and Surgeons yearbook ‘Class Athletes’ section, which showed a male student reaching behind the backs of a female nurse and a female medical student or doctor, intimating sexual assault. Editors conflated the ‘sporting’ theme and sexual predation to facilitate a punchline; captioning the photos ‘Hunting’. It is possible that the women pictured chose to participate in these photos, however, they are positioned turning and walking away from the photographer, seemingly unaware of the male student's gestures behind them. Yearbooks captured a peer culture where bawdy jokes including references to sexual assault were commonplace and trivialised the harassment of women in healthcare.^69^

## Navigating and Confronting Sexual Harassment in Medical Schools

Women’s responses to this misogynistic culture and sexual harassment at medical school, both at the time and in subsequent reflections, were complicated, diverse and often highly individual. In 1975, second-wave feminist concerns about women’s working conditions and violence against women coalesced as activists coined the term “sexual harassment" to describe unwanted sexual behaviour in the workplace. Later, following lawyer Anita Hill’s 1991 testimony about Supreme Court Justice Clarence Thomas and the ruling in Robinson v Jacksonville Shipyards in the same year, legal definitions of sexual harassment extended to include the display of pornographic material in the workplace.^70^ Crucially, even before this legal recognition, many women questioned and articulated their opposition to men’s sexual harassment in work and educational settings.^71^ Some female faculty members published in medical journals, wrote in the popular press, and even testified before congress about sexual harassment in medical school. Their efforts sought to change their colleagues’ behaviour, but also rendered this harassment visible to historians. Other women challenged men’s behaviour in day-to-day interactions; questioning professors about lecture content, objecting to male students’ comments and seeking solidarity with female peers. However, some women faced a backlash for confronting sexual harassment in this way, including experiencing additional misogyny or being pejoratively labelled as ‘a women’s libber’.^72^ Notably, some women doctors did not recall any harassment in subsequent oral histories, perhaps because they did not experience it, see it as noteworthy, or wish to share it. Student publications also suggested the possibility that some women sought incorporation in the dominant peer culture and participated in this sexualised humour. We must also be mindful that this article relies on the testimonies of women who felt comfortable sharing their experiences with historians and journalists, at the time or years later. The historical record is perhaps most likely to exclude or obscure women for whom negative experiences of misogyny and sexual misconduct contributed to their departure from medical training or severely impacted their mental health and safety.^73^

In the 1960s and 1970s, some women doctors and medical students led campaigns to enact feminist change within medicine. In addition to spearheading protests against the pin-up anatomy textbook, Dr Estelle Ramey advocated for women as president of the Association of Women in Science, publicising their plights in *Ms Magazine* and *The Washington Post*.^74^ Other women filed lawsuits, seeking redress and compensation for sexual harassment and discrimination endured at medical schools and in training programmes.^75^ Influenced by the student and civil rights movements of the 1960s, some students advocated for increased diversity among both the student body and faculty. In an oral history, Dr Anita Robinson, who graduated from Jefferson Medical College in 1974, recalled that she created ‘a network of African American students who were in medical schools’ that served as a ‘support system’.^76^ Other coalitions of Black students lobbied for Black faculty members and women campaigned for female appointees.^77^ In 1972, a group of students approached Harvard Medical School to complain that there had never been a woman in the administration and as a result, a committee of female students selected Dr Mary Howell to become the first female Associate Dean. Although in an oral history interview, Howell reflected upon her feelings that the job was tokenistic, she nonetheless used her platform to conduct the research for the pamphlet *Why Would A Girl Go Into Medicine?* and sat on admission panels, where she ‘encouraged the admission of some particular women who didn’t look like the women Harvard thought they wanted’.^78^ Women’s growing representation in medical schools empowered this collective action and fostered further challenges to the mistreatment of female students.

If women protested sexist treatment, however, they could encounter further backlash from male peers and senior doctors. In some cases, male-dominated professional networks spread information about female students, including about their feminist beliefs, which could impact their careers. In 1973, the Stanford Intern Selection Committee reviewed letters of recommendation for female candidates and found that professors praised female students for being feminine. One letter, later quoted in a medical journal, read: ‘Patty is an attractive, mature, personable young lady to have around and who has no “hang-ups” sometimes associated with female physician.’ Likewise, another letter praised a female candidate’s intellect, while highlighting that she avoided being ‘aggressive’ so as ‘not [to] antagonize her male colleagues’.^79^ These letters indicated how gendered prescriptions of behaviour and reactions to discrimination could impact women’s career progression. Female students may have been dissuaded from protesting sexual discrimination and harassment because of concerns it would negatively impact their careers, even as the publication of these letters represented women’s efforts to draw attention to and challenge these discriminatory practices.

Senior women doctors also faced repercussions for challenging the status quo as leading cardiologist Dr Bernadine Healy experienced. An encounter with misogyny at Johns Hopkins University School of Medicine informed her resignation as Professor at that institution, as she detailed in a Washington Post profile and later in her book. In 1982, the Pithotomy Club, an all-male dining society, staged an annual theatrical show; a ‘bonding ritual for male students and faculty, a chance for the once and future elite of American medicine to gather for obscene songs and skits’, as the *Washington Post* explained.^80^ Healy described it as ‘a mean-spirited, X-rated orgy’.^81^ She recalled how ‘originally, the skits were by and about men’, but ‘over time…the show began to target women medical students and physicians’. Healy had a ‘well-known disapproval of the all-male club and its activities’ and she believed that as an act of ‘retaliation’, the 1982 play featured a man posed as Healy ‘dressed in a long blond wig, fish-net stockings and coconut-half brassiere…performing a variety of pornographic acts on other physicians’, before being ‘discovered in flagrante by her ex-husband’. The spectacle ended with a rendition of the 1960 Neil Sedaka’s hit song ‘Calendar Girl’—a song inspired by pin-up photographs of women—with lyrics changed to ‘Cardiology Girl’ in reference to Healy.^82^ Healy reflected that there were ‘no apparent limits on the sexual fantasies acted out at my expense’ and ‘I couldn’t see where pornography fit into a med school curriculum, particularly when made so medical and so personal.’ Healy responded by deciding to ‘give the club a scare’, writing a ‘legal-seeming letter asking for the names of all club members’. While she recalled that the students were ‘quite contrite’, male faculty members ‘implored [Healy] to recognize how important the club was to student life and student-faculty rapport’ and that as that ‘boys will be boys’.^83^ Thereafter, Healy felt isolated in the institution—she recalled, ‘I would go in a room and there were different vibrations’—and she left the university shortly after.^84^ She would go on to hold key positions at the American Red Cross and National Institute of Health, but this experience remained with her.^85^ The 1982 Pithotomy Club production used sexuality to chastise the female professor and restate gendered hierarchies in medicine, which were challenged by women's liberation, feminist women's health politics, and demographic changes in universities.

Although the dominant peer culture stigmatised women, some female M.D.s may have sought to participate in this shared humour. The 1969 Kansas PlayDoc yearbook included a white female member of the graduating class, Morgan, in a centrefold feature that closely followed the *Playboy* template. Photographs showed Morgan posed in an unbuttoned shirt alongside a Playmate-style article. The photographs, commentary on her taste in men, and descriptions of her ‘pleasantly distracting physical characteristics’ arguably objectified Morgan, but the article included her in the shared humour of the editors.^86^ The interview quoted her saying ‘as any girl knows the worst pose is adipose’, meaning fatty tissue.^87^ The comment was both a critique of the female form and witty wordplay, reliant on specialised medical terminology. This display of knowledge and humour incorporated Morgan into the dominant male peer culture. Notably, the yearbook also featured a photograph of a man lying down wearing a pair of boxers and sporting a stethoscope and doctor’s bag.^88^ This yearbook suggested that some women may have navigated this environment by assimilating into this hypersexualised peer culture. Female medical students in the 1960s and 1970s were not only part of a landmark generation, but also individual women at a crucial moment of their lives, where they balanced work and study, friendships, romantic and familial relationships, all while pursuing a medical education. In co-educational settings, female medical students navigated their personal sexuality, while facing sexual harassment. In her oral history interview, Dr Nadine Bruce, a white woman from Illinois, recalled that when she entered the co-educational Illinois Medical School in 1965, having previously attended a women’s college, the presence of male students distracted and excited her. She said that her new awareness led to ‘conflicts within myself’ about how ‘to be popular and date but still be a physician’. Reflecting on these feelings, she later identified this conflict as a struggle in ‘identifying as …[a] woman and a doctor’ in an environment that was a ‘man’s world’.^89^ While Morgan perhaps found her yearbook feature empowering, other women like Bruce were also attempting to navigate their own personal sexuality in an environment that could be characterised by stigma.

In the oral histories I examined, many women doctors denied personally experiencing male students’ hostility, but recalled that sexual jokes or pranks distressed other women. Dr Sarah Sundborg Long, a white woman from Alaska who graduated from Jefferson Medical College in 1970, remembered attending a reduction mammoplasty, a form of breast surgery, as a student. During the procedure, the male surgeon asked her if she would ‘like to hop up on the next table and maybe we can do a transplant?’ In the oral history decades later, she reflected ‘Now you know that might send somebody away…crying’, but she personally found it to be ‘hysterical’, as it remindied her of the ‘banter’ she had encountered growing up and at her parents’ store.^90^ As these reflections suggest, medical students' diverse responses to sexism included minimising these slights or finding humour in them. Dr Nadine Bruce, who graduated from the University of Illinois College of Medicine in 1970, thought that male students welcomed their female classmates; ‘the men in my peer group were so kind…they treated us like their little sisters’. But she did recall ‘one girl who was teased a lot’ as male students ‘would do things which would horrify her’ including one episode where they ‘put rubbers [condoms] on her cadaver’s penis’.^91^ Reflecting on her time as a student, Dr Mary Howell reported ‘some very cruel hazing of some of the prissier women in the class’.^92^ Other women doctors similarly recounted this pattern of harassment; ‘vulnerable ones had like a target on their backs’ and ‘they were picked off by the victimization’.^93^

Meanwhile, other students may have attempted to avoid, ignore or minimise these experiences as a means of self-preservation and survival. Women could share information about particular doctors who mistreated female students, with one woman doctor recalling that ‘you learned who the gropers were and tried to avoid them’.^94^ Other women attempted to minimise adverse experiences. Dr Bernadine Healy reflected that as a medical student in the 1960s; ‘we women ignored the jabs, the lecturers who showed slides of naked women in distinctly nonmedical positions to lighten up a discussion, and the comments that disparaged female patients…because we were grateful to be there’.^95^ In a letter in the *New England Medical Journal*, radiologist Dr Lucy Frank Squire, described how she reframed her experiences of this ‘anti-feminist’ attitude and discrimination as character building, writing that it ‘developed in me work habits and a seriousness of purpose that I might not otherwise have had’.^96^ Squire chose to reinterpret and repurpose these adverse experiences, crediting them with progressing her career, rather than hindering it. Mary Howell criticised this approach, however, describing that ‘the price of character building by way of steady attack on one’s self esteem seems unduly high’.^97^ These varied testimonies show the diversity of women’s reactions to sexual harassment in medical school, which could include overt acts of protest or attempts to minimise or reframe their experiences. They grappled with how sexual harassment impacted them, knowing that their response could in turn impact their future career and wellbeing.

For some women doctors, the experience of reflecting on medical school in oral history interviews many years later altered their perspective on sexual harassment. Dr Linda Lane Izquierdo graduated from Jefferson in 1969 and recalled ‘things that were said to me, later on in residency, that I look back on now and I’m thinking, "Oh my god, sexual harassment!"'. She continued, ‘they were, like, just really not acceptable. But at the time, I remember, I just remember, just accepting it’. In the interview, she stated that she did not want to revisit some of these memories, but during that period she felt she had to ‘adjust to the situation’ because if not ‘you’re not going to last there, you’ll be shocked out of existence and on your way out the door’. Her contemporary Dr Ellen Frank agreed, remembering how she had thought: ‘my job is to survive’.^98^ At this time, male professors, doctors and students attempted to define and defend spaces and definitions of professionalism, which female students internalised, negotiated, confronted and redefined.

## Conclusion

The increasing number of female medical students in the 1960s and 1970s were at once physicians in training, survivors of sexual harassment and discrimination, and individuals with their own sexual agency. When entering medical training, they navigated hypersexualised innuendo and misogyny superimposed on longstanding gendered hierarchies of healthcare work. Male lecturers and students deployed sexual innuendo to bolster a heterosexual, masculine peer culture that they perceived to be imperilled by increasing numbers of women entering medical school and contemporary feminist health politics. This built comradery among male students and faculty, both at the expense of and excluding female students. This learning environment perpetuated gendered and racialised healthcare disparities by promoting these views among the next generation of doctors.

This recourse to sexual jokes and naked photographs discriminated against women by isolating them from the identity of 'doctor' and implying that women’s place in medical school was not as learners, but as sexual objects. As contemporary women’s health campaigners highlighted, and *Playboy* cartoons disturbingly illustrated, this culture contributed to the mistreatment of female patients and other women healthcare professionals. Women medical students responded to these experiences in myriad ways, including minimising or attempting to ignore harassment. Some students challenged their environment by questioning individual instructors and organising collectively to encourage medical school reform, while some female faculty called attention to this mistreatment in articles in leading medical journals and the press. Oral histories indicated that this harassment, including backlash to protests, had long-lasting impacts on women as they worked towards graduation and pursued medical careers thereafter.

Into the twenty-first century, the #metoo movement has further emphasised how sexual violence continues in hospitals and medical schools.^99^ This article has detailed some women’s experiences of sexual harassment, indicating the forms this could take; dirty jokes, comments about women’s bodies, unwanted sexual advances and exposure to sexualised images. The experiences described in this article represent only a fraction of the harassment and discrimination women endured. Sexual harassment was—and continues to be—underreported. This article has indicated the particular barriers medical students and doctors in training face to reporting, including perpetrators’ status within hospital hierarchies and their power over student’s grades and future careers.^100^ For the women who experience sexual harassment during medical school it can have profound, long-lasting impact on their professional and personal lives, as well as their wellbeing.

## Footnotes

1 Howard Saul Becker, ed, *Boys in White: Student Culture in Medical School*, 4th edn (London: Transaction Publishers, 1992), 3–5.

2 Bernadine Healy, *A New Prescription for Women’s Health: Getting the Best Medical Care in a Man’s World* (New York: Viking, 1995), 6.

3 Mary Roth Walsh, *Doctors Wanted: No Women Need Apply: Sexual Barriers in the Medical Profession, 1835–1975* (New Haven: Yale University Press, 1977); Regina Markell Morantz-Sanchez, *Sympathy and Science: Women Physicians in American Medicine* (New York: Oxford University Press, 1985); Darlene Clark Hine, *Hine Sight: Black Women and the Reconstruction of American History* (Bloomington: Indiana University Press, 1994), 147–61.

4 ‘The Late Scene at the Pennsylvania Hospital’, *Philadelphia Evening Bulletin*, 1869; *Pioneers in the Face of Adversity: The Mob of ‘69. Doctor or Doctress? Explore American History Through the Eyes of Women Physicians* (Philadelphia, PA: The Legacy Center, Drexel University College of Medicine Archives & Special Collections), https://doctordoctress.org/islandora/object/islandora:1347, accessed 3 July 2024.

5 Morantz-Sanchez, *Sympathy and Science*, 329.

6 Joan Brumberg and Nancy Tomes, ‘Women in the Professions: A Research Agenda for American Historians’, *Reviews in American History*, 1982, 10, 275–96.

7 American Association of Medical Colleges, ‘Table 1-Medical Students, Selected Years, 1965–2012’, *Women in U.S. Academic Medicine and Science: Statistics and Benchmarking Report 2011–2012*, 2012, 6.

8 Elizabeth C. Patterson, ‘Doctors Wanted: No Women Need Apply: Sexual Barriers in the Medical Profession, 1835–1975/The Hidden Malpractice: How American Medicine Treats Women as Patients and Professionals’, *American Scientist*, 2012, 100, 521.

9 Molly Carnes, ‘What Would Patsy Mink Think?’, *Journal of the American Medical Association*, 2012, 307, 571–72, https://doi.org/10.1001/jama.2012.79.

10 Claudia Goldin and Lawrence Katz, ‘Putting the “co” in Education: Timing, Reasons, and Consequences of College Coeducation from 1835 to the Present’, *Journal of Human Capital*, 2011, 5, 377–417.

11 David M. Carlisle, Jill E. Gardner and Honghu Liu, ‘The Entry of Underrepresented Minority Students into US Medical Schools: An Evaluation of Recent Trends’, *American Journal of Public Health*, 2011, 88, 1314–18.

12 US Bureau of the Census, ‘Detailed Occupation of Employed Persons by Race and Sex for the United States: 1970’, 1973, 3.

13 Gail E. Thomas, ‘Black Higher Education in the 1970s and 1980s: Affirmative Action Implications’, *Educational Policy*, 1988, 2, 137–55.

14 Interviews conducted by Regina Morantz-Sanchez as part of the Oral History Collection on Women in Medicine (1978) project lead by Carol Fenichel, Cynthia S. Pomerleau, Regina Morantz, and Sarah Burke, available via the Women in Medicine Archives and Legacy Centre, Drexel University College of Medicine. Henceforth, referred to as OHCWIM. For more details on the project see Medical College of Pennsylvania, *Oral History Collection on Women in Medicine Catalogue* (1978), https://drexel.edu/~/media/Files/legacy-center/women-in-medicine/a031_findingaid.ashx?la=en. OHCWIM, *Interview with Mary Howell* 15 April 1978, 45. https://drexel.primo.exlibrisgroup.com/view/UniversalViewer/01DRXU_INST/12344621600004721. Also described in Mary Campbell (pseudonym for Mary Howell), ‘Why Would a Girl Go into Medicine?’ (1973), in Barbara Seaman and Laura Eldrifge, eds, *Voices of the Women’s Health Movement**vol. 1* (New York: Seven Stories, 2012), 403.

15 Carrie Pitzulo, *Bachelors and Bunnies: The Sexual Politics of Playboy* (Chicago: University of Chicago Press, 2011), 133.

16 Carrie Pitzulo, ‘The Battle in Every Man’s Bed: “*Playboy*” and the Fiery Feminists’, *Journal of the History of Sexuality*, 2008, 17, 264.

17 Pitzulo, *Bachelors and Bunnies*, 33. Susan Braudy, *Up Against the Centerfold: What It Was Like to Report on Feminism for Playboy in 1969*.

18 Julie Willett, *The Male Chauvinist Pig: A History* (Chapel Hill: University of North Carolina Press, 2021), 76–98.

19 Willett, *The**Male Chauvinist Pig*, 79.

20 Pitzulo, ‘The Battle in Every Man’s Bed’, 259–60.

21 Elizabeth Fraterrigo, *Playboy and the Making of the Good Life**in Modern America* (New York: Oxford University Press, 2009), 176–81.

22 Gloria Steinem, ‘A Bunny’s Tale. Part I’, *Show Magazine*, 1963, 90, 92–94, https://undercover.hosting.nyu.edu/files/original/5c9de8d1db51cede1395f6d6fa480ca24e872b76.pdf.

23 Cartoons from *Playboy*, *Doctor! Doctor!* (London, 1972). A future researcher might look at the depiction of psychiatric consultations, which featured prominently in this collection.

24 Agnes Arnold-Forster, ‘Racing Pulses: Gender, Professionalism and Health Care in Medical Romance Fiction’, *History Workshop Journal*, 2021, 91, 157–81.

25 Joseph Turow, *Playing Doctor: Television, Storytelling, and Medical Power* (New York: Oxford University Press, 1989), 7.

26 Fraterrigo, *Playboy and the Making of the Good Life in Modern America*, 156, 225.

27 Harold I. Kaplan to the *Playboy* Forum, ‘Women Doctors’, *Playboy*, December 1971, 79–80.

28 Frances S. Norris to the *Playboy* Forum, ‘Women in Medicine’, *Playboy*, March 1972, 57.

29 Mary Marvin Johnson to the *Playboy* Forum, ‘Women in Medicine’, *Playboy*, March 1972, 57.

30 Although perhaps a pseudonym ‘John W. Docktor’ was the name of a medical graduate, according to the 1967 Jefferson Medical College yearbook, 145, https://core.ac.uk/download/pdf/155271616.pdf.

31 John W. Docktor to the *Playboy* Forum, ‘Women in Medicine’, *Playboy*, June 1972, 73.

32 R. Vogel, ‘Miss March: *Playboy*’s Playmate of the Month – Nancy Scott’, *Playboy*, March 1964, 88–93; W. Figge, ‘Miss June: *Playboy*’s Playmate of the Month – Kelly Burke’, *Playboy*, June 1966, 96–100.

33 ‘Excuse Me, Nurse’, *Playboy*, November 1968, 224; Chon Day, ‘What’s This Item for’, *Playboy*, October 1969, 250.

34 Jenna Tonn, ‘Why Are We Still Talking About the Naughty Nurse?’, *Lady Science blog*, 17 February 2017, www.ladyscience.com/why-are-we-still-talking-about-the-naughty-nurse/no29.

35 ‘They’re Not Exactly Consulting Physicians’, *Playboy*, April 1965, 12, 166. Another example, ‘God, I Hardly Know Where to Begin!’, *Playboy*, April 1963, 10, 168. ‘Now to Test Your Reflexes’, *Playboy*, March 1969, 16, 213.

36 Alison Bashford, *Purity and Pollution: Gender, Embodiment, and Victorian Medicine* (Basingstoke: Macmillan, 1998), 94–95.

37 Morantz-Sanchez, *Sympathy and Science*, 5.

38 Kim Girouard and Susan Lamb, ‘Scientific Medicine in the Time of Cholera: The Johns Hopkins Ethos and US Friendly Power in North China, 1919’, *European Journal for the History of Medicine and Health*, 2021, 78, 104.

39 Becker, *Boys in White*, 3.

40 Carolyn Decker and Kelsey Duinkerken, ‘Carolyn Parry Decker’, in *First Women at Jefferson Oral Histories*, 2015, 2, https://jdc.jefferson.edu/cgi/viewcontent.cgi?article=1001&context=oral_histories.

41 Linda Izquierdo, Ellen Frank and Kelsey Duinkerken, ‘Kay Ellen Burdette Frank and Linda Lane Izquierdo’, in *First Women at Jefferson Oral Histories*, 2014, 18, https://jdc.jefferson.edu/cgi/viewcontent.cgi?article=1003&context=oral_histories.

42 OHCWIM, *Interview with Nadine Bruce*, 10 December 1977, 22–23, https://drexel.primo.exlibrisgroup.com/view/UniversalViewer/01DRXU_INST/12345101750004721?xywh=209,132,3825,2442&r=0.

43 OHCWIM, *Interview with**Susan Benes*, 10 March 1978, 29, https://drexel.primo.exlibrisgroup.com/view/UniversalViewer/01DRXU_INST/12345535990004721?xywh=770,92,2498,2453&r=0.

44 Healy, *A New Prescription for Women’s Health*, 6.

45 OHCWIM, *Interview with Vanessa Gamble*, 15 March 1978, 34-5, https://drexel.primo.exlibrisgroup.com/view/UniversalViewer/01DRXU_INST/12345536010004721?xywh=null&r=0. See also OHCWIM, *Interview with**Victoria Nichols*, 24 March 1978, 26, https://drexel.primo.exlibrisgroup.com/view/UniversalViewer/01DRXU_INST/12345345860004721?c=&m=&s=&cv=&xywh=-2602,-251,8688,5010&r=0

46 OHCWIM, *Interview with Vanessa Gamble*, 31.

47 'Dr Stephanie Joan Woolhandler', *Changing the Face of Medicine,*cfmedicine.nlm.nih.gov/physicians/biography_335.html, accessed 3 July 2024. Dr Linda Lane Izquierdo and Ellen Frank who graduated from Jefferson Medical College in 1969 also recalled the use of nude images of women in lectures in a 2014 oral history. Linda Izquierdo, Ellen Frank and Kelsey Duinkerken, ‘Kay Ellen Burdette Frank and Linda Lane Izquierdo’, in *First Women at Jefferson Oral Histories*, 2014, 6, https://jdc.jefferson.edu/oral_histories/1.

48 Campbell, 'Why Would a Girl Go into Medicine?'.

49
*Ibid.*, 405.

50 Mia Warren, Danielle Roth, Isabel Dobrin, "For These 3 Women, Medical Careers Are A Family Affair", *National Public Radio,* 26 January 2018, https://www.npr.org/2018/01/26/580694221/for-these-3-women-medical-careers-are-a-family-affair.

51 On anatomy education and Black athletes see Patrick B. Miller, ‘The Anatomy of Scientific Racism: Racialist Responses to Black Athletic Achievement’, *Journal of Sport History*, 1998, 25, 119–51; In particular 131–32.

52 Warren et al, ‘For These 3 Women’.

53 Roland Frederick Becker, James Walter Wilson and John A. Gehweiler. *The Anatomical Basis of Medical Practice* (Baltimore: Williams and Wilkins, 1971).

54 Amanda M. Friz and Marissa L. Fernholz, ‘The Male Gaze in the Medical Classroom: Proximity, Objectivity, and Objectification in "The Pornographic Anatomy Book"', *Women’s Studies in Communication*, 2020, 43, 292–316; Patricia Louie and Rima Wilkes, ‘Representations of Race and Skin Tone in Medical Textbook Imagery’, *Social Science & Medicine*, 2018, 202, 38–42; Cameron A. Lynch, ‘Diversifying Medical School Education to Represent BAME Backgrounds’, *British Medical Journal*, 2020, 370.

55 Edward C. Halperin, ‘The Pornographic Anatomy Book? The Curious Tale of the Anatomical Basis of Medical Practice’, *Academic Medicine: Journal of the Association of American Medical Colleges*, 2009, 84, 278–83.

56 Estelle Ramey Speaking before US Congress, Committee on Education and Labor, *The Women’s Educational Equity Act Hearings, 93rd Cong., 1st Sess.* (Washington: U.S. Govt. Print. Off., 1973), 493–503.

57 Campbell, 'Why Would a Girl Go into Medicine?', 403.

58 Healy, *A New Prescription for Women’s Health*, 8.

59 Sarah Aiston, ‘“A Woman’s Place”: Male Representations of University Women in the Student Press of the University of Liverpool, 1944–1979’, *Women’s History Review*, 2006, 15, 3–34.

60 University of Utah School of Medicine, *Medicine Man Yearbook,* 1973, 38, Spencer S. Eccles Health Sciences Library, University of Utah, https://collections.lib.utah.edu/details?id=2434419.

61 University of Kansas Medical School, Jayhawker Yearbook *PlayDoc*, 1969, 152–90. I am indebted to the historian of medicine and photography Brandon Zimmerman, who identified the *Playboy* themes in the 1969 Kansas and Jefferson yearbooks. Our early discussions at the Women in Medicine Legacy Center and Archives at Drexel had a formative influence on the development of this research and my understanding of visual culture and medical education more broadly.

62
*Jefferson Medical College Yearbook*, 1969. Archives & Special Collections, Thomas Jefferson University.

63 ‘An Interview with Dr Estelle Ramey’, 1971, quoted in Franz Ingelfinger, ‘Doctor Women’, *The New England Journal of Medicine*, 1974, 291, 303–4; OHCWIM, *Interview with Mary Howell*, 24.

64 OHCWIM, *Interview with Mary Howell* (1977), 24; Anne Walling, Kari Nilsen and Kimberly J. Templeton, ‘The Only Woman in the Room: Oral Histories of Senior Women Physicians in a Midwestern City’, *Women’s Health Reports*, 2020, 283.

65 Columbia University College of Physicians and Surgeons, *Physicians and Surgeons* (1969), 55, Archives & Special Collections, Columbia University Health Sciences Library, https://archive.org/details/psyearbookofcoll1969colu/page/54/mode/2up; OHCWIM, *Interview with Judith Herman,* 26–27, https://drexel.primo.exlibrisgroup.com/view/UniversalViewer/01DRXU_INST/12344996280004721.

66 OHCWIM, *Interview with Judith Herman*, 26-7.

67
*Ibid*, 27.

68 Walling et al., ‘The Only Woman in the Room’, 283.

69 Columbia University College of Physicians and Surgeons, *1968 Yearbook: Physicians and Surgeons* (1969), 87, Archives & Special Collections, Columbia University Health Sciences Library, https://archive.org/details/psyearbookofcoll1969colu/page/86/mode/2up.

70 Nell J. Medlin, ‘Expanding the Law of Sexual Harassment to Include Workplace Pornography: Robinson v. Jacksonville Shipyards, Inc.’, *Stetson Law Review*, 1992, 21, 661, 655–80; Tara R. Price, ‘You’ve Come a Long Way, Baby: Stripping Pornography from America’s Workplace: Robinson v. Jacksonville Shipyards, Inc., 760 F. Supp. 1486 (M.D. Fla. 1991)’, *The Florida Historical Quarterly*, 2013, 92, 261–75.

71 Carrie N. Baker, *The Women’s Movement Against Sexual Harassment* (Cambridge: Cambridge University Press, 2007), 11–26.

72 Campbell, 'Why Would A Girl Go Into Medicine?'.

73 Walling et al., ‘The Only Woman in the Room’, 286.

74 Elaine Woo, ‘Estelle Ramey, 89; Doctor, Sharp-Tongued Feminist’, *Los Angeles Times*, 17 September 2016, https://www.latimes.com/archives/la-xpm-2006-sep-17-me-ramey17-story.html.

75 Dr Annabelle Lipsett brought a legal challenge against the University of Puerto Rico because of the gender discrimination and sexual harassment she experienced as a surgical resident in the early 1980s.

76 Anita Robinson and Kelsey Duinkerken, ‘Anita Robinson’, in *First Women at Jefferson Oral Histories* (2015), 2, https://jdc.jefferson.edu/oral_histories/10/.

77 The Black Medical Students of UCLA, ‘Letter from Black Medical Students of UCLA, 1970’, https://calstatela-exhibits.libraryhost.com/items/show/18.

78 OHCWIM, *Interview with Mary Howell*, 42, 45.

79 Dr Sonia Bauer, a member of the selection committee, published these extracts in a letter in the *New England Journal of Medicine*, 1974, 291, 1141.

80 ‘Pithotomy’ is a Greek term that translates approximately to tapping a keg of beer. The society was comprised only of men from its foundation by the class of 1897 until 1990; it dissolved two years later. William H. Jarrett ‘The Pithotomy Club: RIP’, *Baylor University Medical Center Proceedings*, 2011, 24, 35–41; Malcolm Gladwell, ‘The Healy Experiment’, *The Washington Post*, 21 June 1992, www.washingtonpost.com/archive/lifestyle/magazine/1992/06/21/the-healy-experiment/be3fd99e-aec1-4ddd-84bc-a04ed02a4faa/.

81 Healy, *A New Prescription for Women’s Health*, 7.

82 Gladwell, ‘The Healy Experiment’.

83 Healy, *A New Prescription for Women’s Health*, 7. Kimberly L. Limbrick ‘Developing a Viable Cause of Action for Student Victims of Sexual Harassment: A Look at Medical Schools’, *Maryland Law Review,* 1995, 54, 601–32.

84 Gladwell, ‘The Healy Experiment’.

85 Healy, *A New Prescription for Women’s Health*, 7.

86 Laura Mulvey, ‘Visual Pleasure and Narrative Cinema’, *Screen*, 1975, 16, 6–18.

87 University of Kansas Medical School, Jayhawker Yearbook *PlayDoc*, 1969. Notably, in a lawsuit brought by Charlotte Horowitz against the University of Missouri, Kansas City, it was reported that during her time as a medical student from 1970 to 1972, she faced fatphobia intersecting with gender discrimination and antisemitism, see Yale L. Rosenberg, ‘The Horowitz Affair’, *Boston University Law Review*, 1978, 58, 5, 738.

88 The male model is not identified by name or role as a student or other medical professional.

89 OHCWIM, *Interview with Nadine Bruce,* 23.

90 Sarah Long, Marie Russell and Kelsey Duinkerken, ‘Marie Olivieri Russell and Sarah Sundborg Long’, in *First Women at Jefferson Oral Histories* (2015), 11, https://jdc.jefferson.edu/cgi/viewcontent.cgi?article=1010&context=oral_histories.

91 OHCWIM, *Interview with Nadine Bruce* , 23–24.

92 OHCWIM, *Interview with Mary Howell*, 23–24.

93 Walling, ‘The Only Woman in the Room’, 283.

94
*Ibid.*

95 Healy, *A New Prescription for Women’s Health*, 6.

96 Lucy Frank Squire to the Editor, *New England Medical Journal,* 1974, 291, 1139.

97 Mary Howell to the Editor, *Ibid.*, 1141.

98 ‘Kay Ellen Burdette Frank and Linda Lane Izquierdo’, 23–24.

99 Arghavan Salles, ‘Sexual Harassment Is Still the Norm in Health Care’, *Scientific American**blog*, 24 October 2019, https://www.scientificamerican.com/blog/voices/sexual-harassment-is-still-the-norm-in-health-care/; Esther K. Choo et al., ‘From #MeToo to #TimesUp in Health Care: Can a Culture of Accountability End Inequity and Harassment?’, *The Lancet*, 2019, 393, 499–502; Margaret Cronin Fisk, ‘America’s Medical Profession Has a Sexual Harassment Problem’, 30 April 2019, https://www.bloomberg.com/news/features/2019-04-30/america-s-medical-profession-has-a-sexual-harassment-problem; Simon Fleming and Rebecca A. Fisher, ‘Sexual Assault in Surgery: A Painful Truth’, *The Bulletin of the Royal College of Surgeons of England*, 2021, 103, 282–85.

100 Delese Wear and Julie Aultman, ‘Sexual Harassment in Academic Medicine: Persistence, Non-reporting, and Institutional Response’, *Medical Education Online*, 2005, 10, 1, https://doi.org/10.3402/meo.v10i.4377.

## Notes

Elizabeth Evens (she/her) is a medical student at Newcastle University, UK. She holds a PhD from University College London, where her project considered the history of gender, medicine, and policing. She received the 2019 Roy Porter Essay Prize from the Society for the Social History of Medicine.

